# Supplementary material for: Molecular basis for polysaccharide recognition and modulated ATP hydrolysis by the O antigen ABC transporter
Source: Nat Commun. 2022 Sep 5;13:5226. doi: 10.1038/s41467-022-32597-2 (PMC9445017; doi:10.1038/s41467-022-32597-2)
Supplement: Supplementary file 1 — Supplementary Information [file 41467_2022_32597_MOESM1_ESM.pdf]

Molecular basis for polysaccharide recognition and modulated ATP hydrolysis by the O antigen ABC transporter

Nicholas Spellmon[1,2], Artur Muszyński[3], Ireneusz Górniak[1,2], Jiri Vlach[3], David Hahn[3], Parastoo Azadi[3], Jochen Zimmer*[1,2]

Affiliations:

[1]Howard Hughes Medical Institute, University of Virginia School of Medicine

[2]Department of Molecular Physiology and Biological Physics, University of Virginia School of Medicine, Charlottesville, VA, 22903, USA

[3]Complex Carbohydrate Research Center, University of Georgia, Athens, GA, 30602, USA

* Corresponding author: jz3x@virginia.edu

Supplementary Information

Intact LPS	
**Glycosyl residue**	**Mole %**
Me-Rha	0.1*
Rha	72.3
GalA	7.4
Me-Man	0.3*
Man	11.7
Gal	1.8
Glc	0.4
Hep	4.2
Kdo	1.3
GlcNAc	0.7

Supplementary Table 1| GC-MS glycosyl composition of *A. aeolicus* intact LPS used in this work. The LPS was methanolysed, re-N-acetylated and converted to trimethylsilyl (TMS) methyl glycosides.
* Due to unavailability of the authentic standards of methylated Man and methylated Gal, we used response factors corresponding to the unsubstituted Rha and Man standards. Thus, these values only represent approximate mole %.

Residue† (linkage)	Atom	Group						
		1	2	3	4	5	6(a,b)	*O*-Me
**Aa**	^{1}H	5.20	4.23*	3.90	3.70	3.78	3.86,3.77	
→2)-α-D-Man*p*(1→	^{13}C	102.65	79.9	72.4	69.8	76.0	63.4	
**Ab**	^{1}H	5.19	4.22	3.90	3.70	3.78	3.86,3.77	
→2)-α-D-Man*p*(1→	^{13}C	102.70	80.0	72.4	69.8	76.0	63.4	
**Ba**	^{1}H	5.13	4.23	3.86	3.47	3.85	1.28	
→2)-α-D-Rha*p*(1→	^{13}C	102.55	79.9	72.1	75.1	71.9	19.3	
**Bb**	^{1}H	5.12	4.22	3.86	3.47	3.85	1.28	
→2)-α-D-Rha*p*(1→	^{13}C	102.58	80.0	72.1	75.1	71.9	19.3	
**Ca**	^{1}H	4.73	4.16	3.69	3.49	3.43	1.32	
→3)-β- D-Rha*p*(1→	^{13}C	101.0	73.3	82.9	73.9	74.8	19.4	
**Cb**	^{1}H	4.72	4.15	3.69	3.49	3.43	1.32	
→3)-β- D-Rha*p*(1→	^{13}C	100.9	73.4	82.9	73.9	74.8	19.4	
**Da**	^{1}H	4.73	4.10	3.65	3.48	3.43	1.32	
→3)-β- D-Rha*p*(1→	^{13}C	101.0	73.4	83.2	73.9	74.8	19.4	
**Db**	^{1}H	4.72	4.09	3.65	3.48	3.43	1.32	
→3)-β- D-Rha*p*(1→	^{13}C	100.9	73.5	83.2	73.9	74.8	19.4	
**F**	^{1}H	5.14	4.30	3.60	3.69	3.79	n.d.	3.45
t-α-Man(3OMe)(1→	^{13}C	104.8	68.6	82.4	68.4	75.9	n.d.	59.0
**G**	^{1}H	5.06	4.30	3.55	3.48	3.85	1.28	3.44
t-α-Rha(3OMe)(1→	^{13}C	104.9	68.6	82.1	73.7	71.9	19.3	58.9

Supplementary Table S2| ^{1}H and ^{13}C chemical shifts (ppm) of residues forming the HMW *O* polysaccharide polymer.
***** The underlined chemical shifts belong to groups involved in glycosidic bond formation.
† The intra-chain residues were assigned the D-configuration according to the results of absolute configuration analysis.
n.d., not determined

Signal	Area (10^6, a.u.)	Normalized area
Aa	1.81	25.2
Ab	1.25	17.3
Ba	2.93	40.9
Bb	1.46	20.4
Ca+Da	2.80	38.9
Cb+Db	4.39	61.1

Supplementary Table S3| Deconvoluted ^{1}H integral intensities of anomeric signals. Normalized areas were calculated so that the area of C+D = 100.0.

WztC & 3-O-methyl-D-mannose

**Data collection**	**8DKY**	
Space group	$P2_12_12_1$	P1
Wavelength (Å)	1.0	1.0
Cell dimensions		
a, *b*, *c* (Å)	56.56 58.17 95.32	56.44 58.02 95.17
α, β, γ (°)	90 90 90	89.92 89.96 89.82
Resolution (Å)	95.32 - 1.61 (1.64 - 1.61)	95.17 – 1.75 (1.78 – 1.75)
R_{merge}	0.087 (0.782)	0.136 (0.733)
R_{pim}	0.037 (0.355)	0.064 (0.476)
$CC_{1/2}$	0.997 (0.809)	0.981 (0.714)
$I / \sigma I$	12.0 (2.0)	4.8 (1.5)
Completeness (%)	99.3 (91.7)	90.8 (92.3)
Redundancy	6.8 (5.6)	3.0 (3.2)
**Refinement**		
Resolution (Å)	40.55 - 1.61	
No. reflections		
Total	41093 (3888)	
R_{free}	2125 (187)	
R_{work} / R_{free}	0.2598 / 0.2872	
No. atoms		
Protein	2344	
Man3Me	26	
Water	152	
B-factors (Å^2)		
Chain A	26.74	
Chain B	25.30	
Man3Me A	26.54	
Man3Me B	24.31	
Water	31.62	
R.m.s. deviations		
Bond lengths (Å)	0.005	
Bond angles (°)	0.95	

Values in parentheses are for the highest-resolution shell

Supplementary Table 4| X-ray data collection and refinement.

	WzmWzt (POPG ND)	WzmWzt ADP (POPG ND)	WzmWzt OPS (E.coli lipid ND)	WzmWzt Man3Me (E.coli lipid ND)	WzmWzt LPS (E.coli lipid ND)	WzmWzt ADP (E.coli lipid ND)	WzmWzt ATP (E.coli lipid ND)	WzmWzt (DDM)
**Data Collection**		8DOU, EMD-27623	8DKU, EMD-27491	8DN8, EMD-27556		8DNC, EMD-27563	8DNE, EMD-27564	8DL0, EMD-27494
Microscope	Titan Krios	Titan Krios	Titan Krios	Titan Krios	Titan Krios	Titan Krios		Titan Krios
Camera	K3	K3	K3	K3	K3	K3		K3
Magnification	81000x	81000x	81000x	81000x	81000x	81,000x		81000x
Voltage (kV)	300	300	300	300	300	300		300
Electron dose ($e^-/Å^2$)	50	50	50	52	50	50		50
Defocus Range (μm)	-2.0 to -1.0	-2.0 to -1.0	-2.0 to -1.0	-2.0 to -1.0	-2.0 to -1.2	2.0 to -1.0		-2.0 to -1.0
Pixel Size	1.08	1.12	1.08	1.08	1.08	1.08		1.08
**Data Processing**								
Symmetry imposed	C1	C1	C1	C1	C1	C1		C1
Initial particles (#)	1818546	4288892	4019981	355654	5449466	6164555		1438647
Final particles (#)	167834	356190	410460	91999	264525	443584	139043	105151
Map Resolution (Å)	3.65	3.63	3.24	3.74	3.27	3.26	3.52	4.05
FSC threshold	0.143	0.143	0.143	0.143	0.143	0.143	0.143	0.143
**Refinement**								
Initial Model Used		7K2T	7K2T, 8DKY	8DKU		8DKU	7K2T	6M96, 8DKY
Model resolution (Å)		3.5	3.2	3.7		3.3	3.5	4.1
Map sharpening		-143.1	-153.7	-145.7		-135.5	-112.5	-134.6
**Model Composition**								
Non-hydrogen atoms		10670	10644	10638		10672	10286	10377
Protein residues		1300	1300	1300		1300	1252	1268
Ligands		2	1	2		3	4	0
***B* factors ($Å^2$)**								
Protein		41.22	52.64	81.44		47.61	70.57	23.26
Ligand		41.02	66.78	155.39		53.75	43.48	-
**R.m.s deviations**								
Bond lengths		0.02	0.004	0.003		0.004	0.003	0.003
Bond Angles		0.546	0.623	0.604		0.641	0.652	0.662
**Validation**								
MolProbity Score		1.69	1.81	1.94		1.93	1.84	1.91
Clashscore		6.73	7.49	9.82		9.57	9.42	13.19
Rotamer outliers (%)		0.17	0	0		0	0	0
**Ramachandran plot**								
Favored (%)		95.36	94.04	93.96		93.50	95.06	95.94
Allowed (%)		4.64	5.96	6.04		6.5	4.94	4.06
Disallowed (%)		0	0	0		0	0	0
**Model vs. Data**								
CC mask		0.76	0.74	0.74		0.74	0.78	0.61
CC box		0.70	0.66	0.69		0.68	0.71	0.65
CC peaks		0.63	0.62	0.60		0.62	0.62	0.50
CC volume		0.73	0.72	0.74		0.73	0.75	0.65
Mean CC for ligands		0.73	0.74	0.64		0.78	0.86	-

Supplementary Table 5| Cryo-EM data collection and refinement

Supplementary Methods: Structure determination of O antigen polysaccharide by NMR

The ^{1}H and ^{1}H,^{13}C-HSQC NMR spectra of *A. aeolicus* O polysaccharide released from HMW LPS contained six distinct anomeric signals (H1/C1) resonating at (ppm): 5.20/102.65, 5.19/102.7, 5.13/102.55, 5.12/102.58, 4.73/101.0, and 4.72/100.9. As explained in detail below, the six anomeric signals in fact originated from eight distinct residues, owing to signal overlaps (Supplementary Table 2). In addition to these strong signals, we observed a set of minor signals of anomeric protons and carbons likely due to low content of the core oligosaccharide.

Analysis of the 1D ^{1}H and a set of 2D NMR experiments (COSY, TOCSY, NOESY, ^{1}H,^{13}C-HSQC and ^{1}H,^{13}C-HMBC) provided complete assignments of ^{1}H and ^{13}C resonances of the major residues (Supplementary Table 2). An overview of correlation signals instrumental in obtaining the general signal assignments and residue linkages is given in Figure S1A. Based on the chemical shifts of individual sugar residues, and in agreement with the chemical analyses, we found that the two partly overlapping anomeric signals at 5.20/5.19 ppm belong to two 2-substituted α-D-Man*p* residues [→2)-α-D-Man*p*(1→; residues Aa and Ab] whose signals of CH groups 3–6 were indistinguishable. Similarly, the two partly overlapping anomeric signals at 5.13/5.12 ppm belong to two 2-substituted α-D-Rha*p* residues [→2)-α-D-Rha*p*(1→; residues Ba and Bb] whose signals of groups 3–6 completely overlapped. The partly overlapping anomeric signals at 4.73 and 4.72 ppm belong to 3-substituted β-D-Rha*p* residues [→3)-β-D-Rha*p*(1→] that have resolved signals of CH groups 2 and 3 but overlapping signals of groups 4–6. These two anomeric signals were originally designated as belonging to two residues C and D. However, as explained below, this group of signals consisted of four peaks belonging to residues Ca, Cb, Da and Db, where the anomeric signals of Ca and Da, as well as Cb and Db coincided.

The NOESY and HMBC spectra (Supplementary Fig.1a) showed that residues A are linked to the 3-position of residues C, while residues B are linked to the 3-position of residues D, thus forming A–C and B–D pairs. Residues C and D were clearly linked to the 2-positions of residues A or B; however, due to nearly complete overlap of A2 and B2 signals, it was not possible to determine directly which of the four possible linkages were present (C–A, C–B, D–A and/or D–B).

Several NOESY signals (Supplementary Fig.1b) were critical in establishing that residue C gave two distinct sets of signals (labeled Ca and Cb), and similarly for residue D (Da and Db). The NOEs between C1 and C2 groups, as well as D1 and D2 groups, appeared at both anomeric frequencies 4.73 and 4.72 ppm (shown in red in Supplementary Fig.1b). The only explanation for this observation was the presence of two slightly non-equivalent residues Ca and Cb and two non-equivalent residues Da and Db, whereby the anomeric signals of Ca and Da overlap and so do the anomeric signals of Cb and Db. Similar C1–C2 and D1–D2 pairs of correlation signals are seen in the TOCSY and COSY spectra (not shown), confirming the assignments.

The mode of involvement of the two C and two D residues in the sequence became clear from two weak NOESY signals observed between Aa1 and Da2, and between Bb1 and Cb2 (shown in red in Supplementary Fig.1b). Such NOEs are only possible if residue Da is linked to residue Aa, and residue Cb to residue Bb. Consequently, and in concert with all the other correlations seen in the NOESY spectrum, residue Db is linked to residue Ba, and residue Ca to residue Ab. The whole situation is illustrated in Figure S1C-a.

The structural findings were corroborated and expanded by the analysis of ^{1}H integral intensities. The collective integral intensities of the anomeric signals were in the ratio of 2:3:5 for A:B:(C+D), which means the A–C and B–D pairs are in 2:3 molar ratio, *i.e.* the empirical formula of the polymer is $(AC)_2(BD)_3$ (Supplementary Fig.1c-b). Deconvolution of the partially overlapping signals afforded approximate integral intensities of the individual

a/b peaks (Supplementary Table 3). From the integrals, the Aa:Ab ratio was about 25:17, which can be reduced to 3:2; the Ba:Bb ratio about 2:1, and (Ca+Da):(Cb+Db) ratio 2:3.

Based on the above NOE analysis of residue connectivities, we can deduce that Aa:Ab = Da:Ca and Ba:Bb = Db:Cb. The smallest integer molar ratio of the residues that satisfies all the above constrains is Aa:Ab:Ba:Bb = Da:Ca:Db:Cb = 6:4:10:5. However, when a summary structure is produced using the 10:5 = 2:1 (Ba:Bb) ratio, some of the other residues are no longer in the 2:3 ratios, and also the overall 2:3 ratio of A–C and B–D subunits is perturbed (Supplementary Fig.1C-c). Specifically, there is one extraneous residue Db, and one missing residue Cb. Instead, it is possible that the true Ba:Bb ratio is 3:2 and that the somewhat higher value of 2:1 was obtained due to imperfect deconvolution. Assuming Ba:Bb ratio of 3:2, the summary structure is as shown in Figure S1C-d, satisfying the overall 2:3 ratio. The pairs of residues in the summary structure in Supplementary Fig.1c-4 were further recombined to obtain the smallest number of longest fragments that consist of the appropriate A–C and B–D pairs. The condensed structure, also shown in color in Figure S1C-e, is thus $(DbBa)_9(DaAaCbBb)_2(DaAaCaAbCbBb)_4$. The three fragments encompassed in the brackets can be freely repositioned and any such structure satisfies all the constraints that were outlined above. We acknowledge that this is an idealized structure and does not account the limited length of the polymer and the presence of terminal groups. These factors may have influenced the higher-than 3:2 Ba:Bb ratio suggested by the deconvolution.

Two of the minor anomeric signals were found to belong to 3-*O* methylated mannose and rhamnose residues that formed the non-reducing end of the O-chain polymer. The residues were identified based on nearly complete assignments of their chemical shifts (Supplementary Table 2) that were obtained from the same NMR spectra used for the O-chain structure determination. The HMBC spectrum showed that the terminal residue F [3-*O*-Me-α-Man*p*(1→] is linked to the 3-position of a residue similar to residue C, while the terminal residue G [3-*O*-Me-α-Rha*p*(1→] is linked to the 3-position of a residue similar to residue D. Thus, the residues F and G resemble the polymer residues A and B, respectively.

SUPPLEMENTARY FIGURES AND LEGENDS

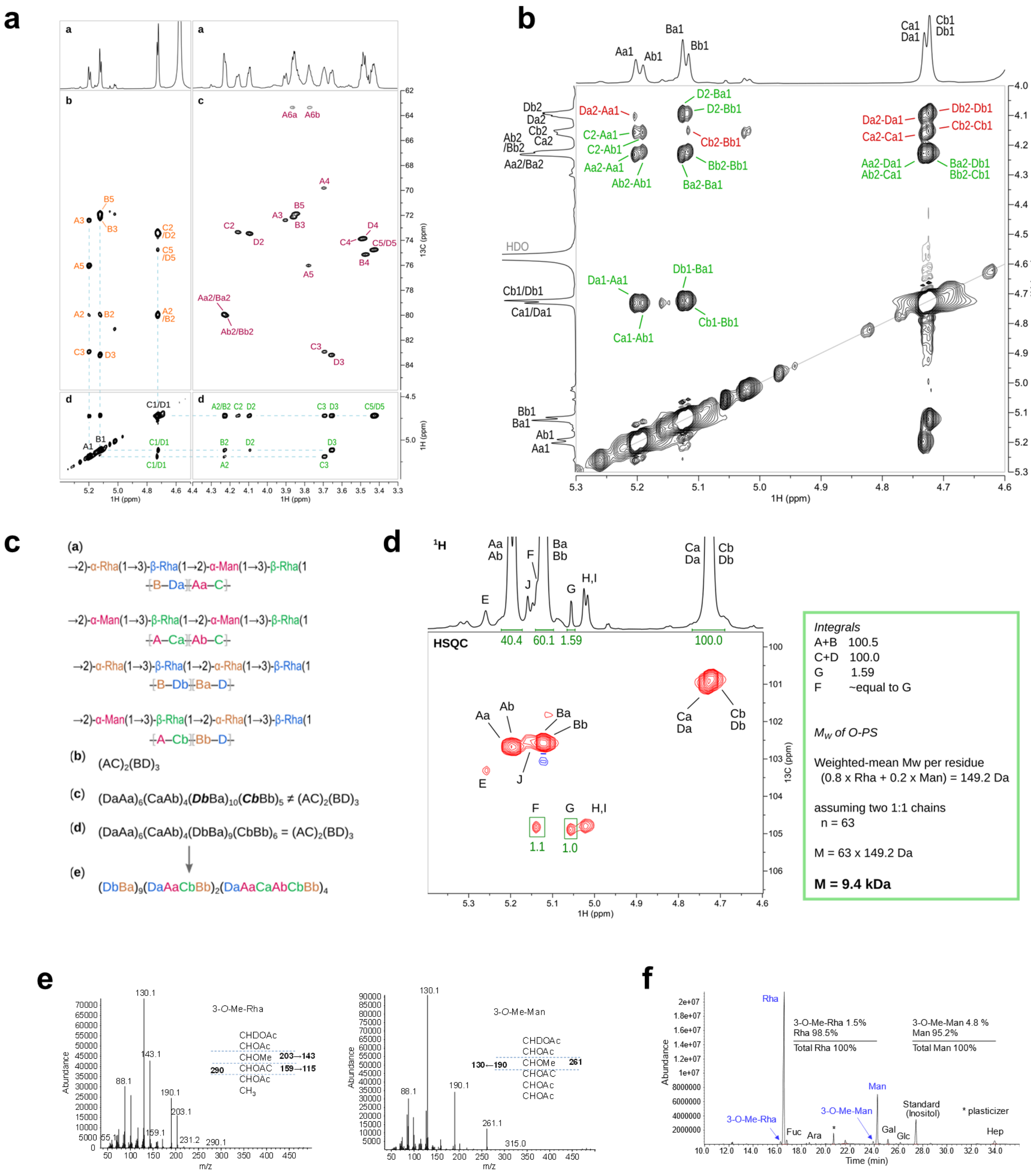

Supplementary Fig. 1| Structural motifs and estimated size of the *A. aeolicus* O antigen. a, Regions of the *A. aeolicus* HMW O antigen spectra showing signals instrumental in obtaining general assignments and linkage information, without distinguishing the a/b subsets of the residues. (a) ^{1}H NMR spectrum, (b) $^{1}H,^{13}C$-HMBC spectrum, (c) $^{1}H,^{13}C$-HSQC spectrum (black: positive contours, gray: negative contours), (d) $^{1}H,^{1}H$-NOESY spectrum. In HMBC, the f1 ^{13}C assignments for peaks correlating with the anomeric signals are shown in orange. In NOESY, the f2 ^{1}H assignments for cross-peaks originating from the anomeric signals are shown in green.

b, A region of the 2D NOESY spectrum of HMW O polysaccharide showing detailed assignments of NOEs between the H1 and H2 signals for all major residues. The signals important for structure elucidation are labeled in red. A 1D ^{1}H spectrum is also shown with signal assignments along the horizontal and vertical borders. **c**, Identification of structural motifs present in the *A. aeolicus* HMW OPS. **d**, Molecular weight estimation of the O antigen based on the ^{1}H signal integrals of the main-chain residues A–D and the resolved signal of terminal residue G. The integrals of HSQC signals of F and G show that the terminal residues are approximately equimolar. The peaks that likely belong to the adaptor-core region are labeled E, H, I and J. The calculation of the molecular weight of the O-chain polymer is outlined in the panel on the right. **e**, EI-MS spectra and fragmentation of 3-*O*-Me-Rha (left) and 3-*O*-Me-Man (right). **f**, GC-MS chromatogram of the OPS converted to alditol acetates. Highlighted is the % distribution of the EI peak of Rha vs. 3-*O*-Me-Rha and the % distribution of the EI peak of Man vs. 3-*O*-Me-Man.

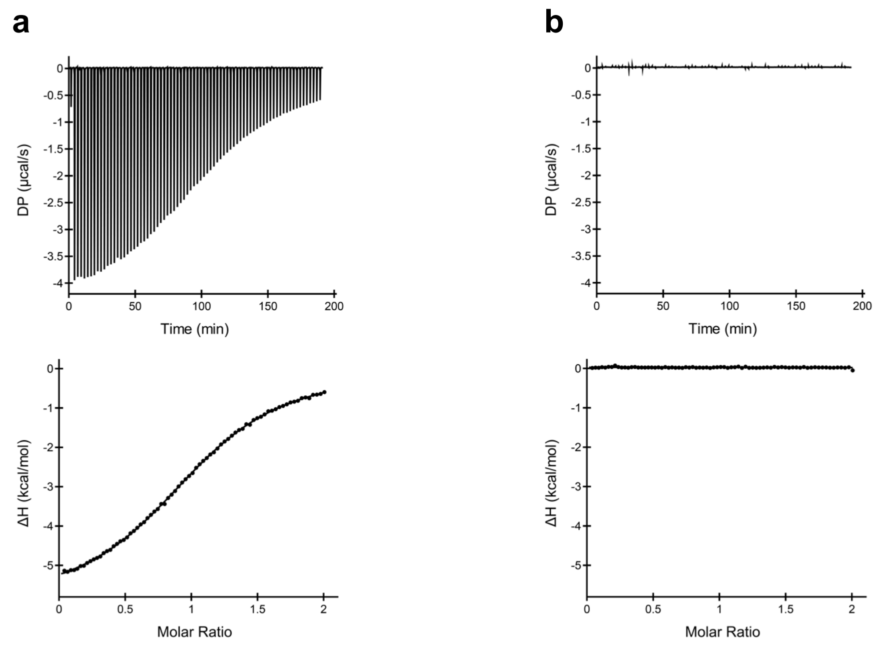

c

Ligand	N	K_D (M)	ΔH (kcal/mol)	ΔG (kcal/mol)	-TΔS (kcal/mol)
3-O-Me-D-Man	1.09	2.34E-04	-6.01	-4.95	1.06
D-Mannose	N.D.	N.D.	N.D.	N.D.	N.D.

Abbreviations: D-Man3Me, 3-O-methyl-D-mannose; K_D, dissociation constant; N.D., no binding detected

Supplementary Fig. 2| Isothermal Titration Calorimetry of Wzt-CBM binding to 3-O-methyl-D-mannose or D-mannose. a, Raw data (top) and isotherm plot (bottom) for Wzt-CBM and 3-O-methyl-D-mannose. **b**, Raw data (top) and isotherm plot (bottom) for Wzt-CBM and D-mannose. **c**, Thermodynamic parameters obtained from fitting the isotherm plot into a one-site binding model.

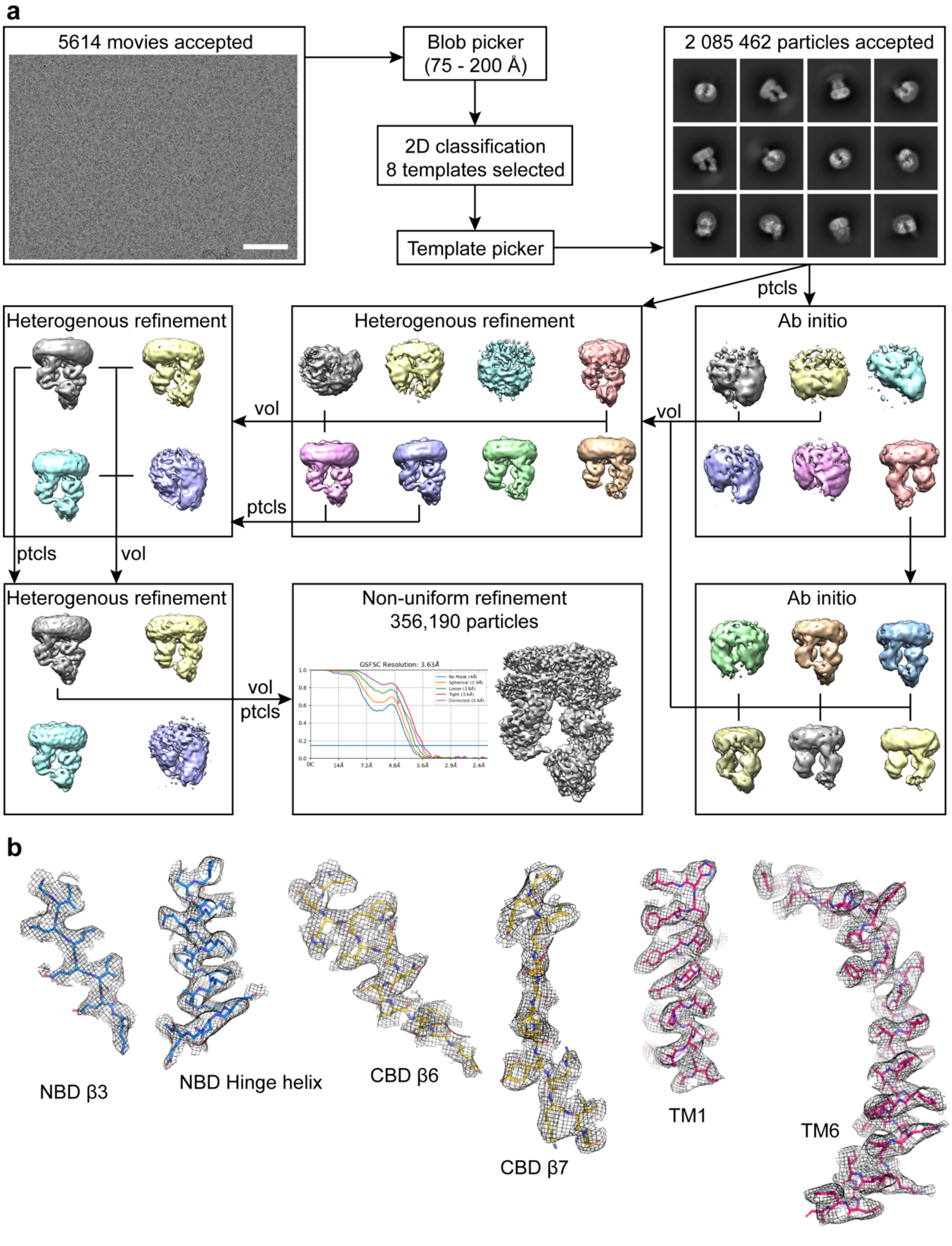

Supplementary Fig. 3| Cryo-EM data processing of ADP-bound WzmWzt. a, Cryo-EM workflow of the ADP-bound/apo WzmWzt data sets. Scale bar on EM image: 100 nm. **b**, Examples of EM map quality contoured at 5σ.

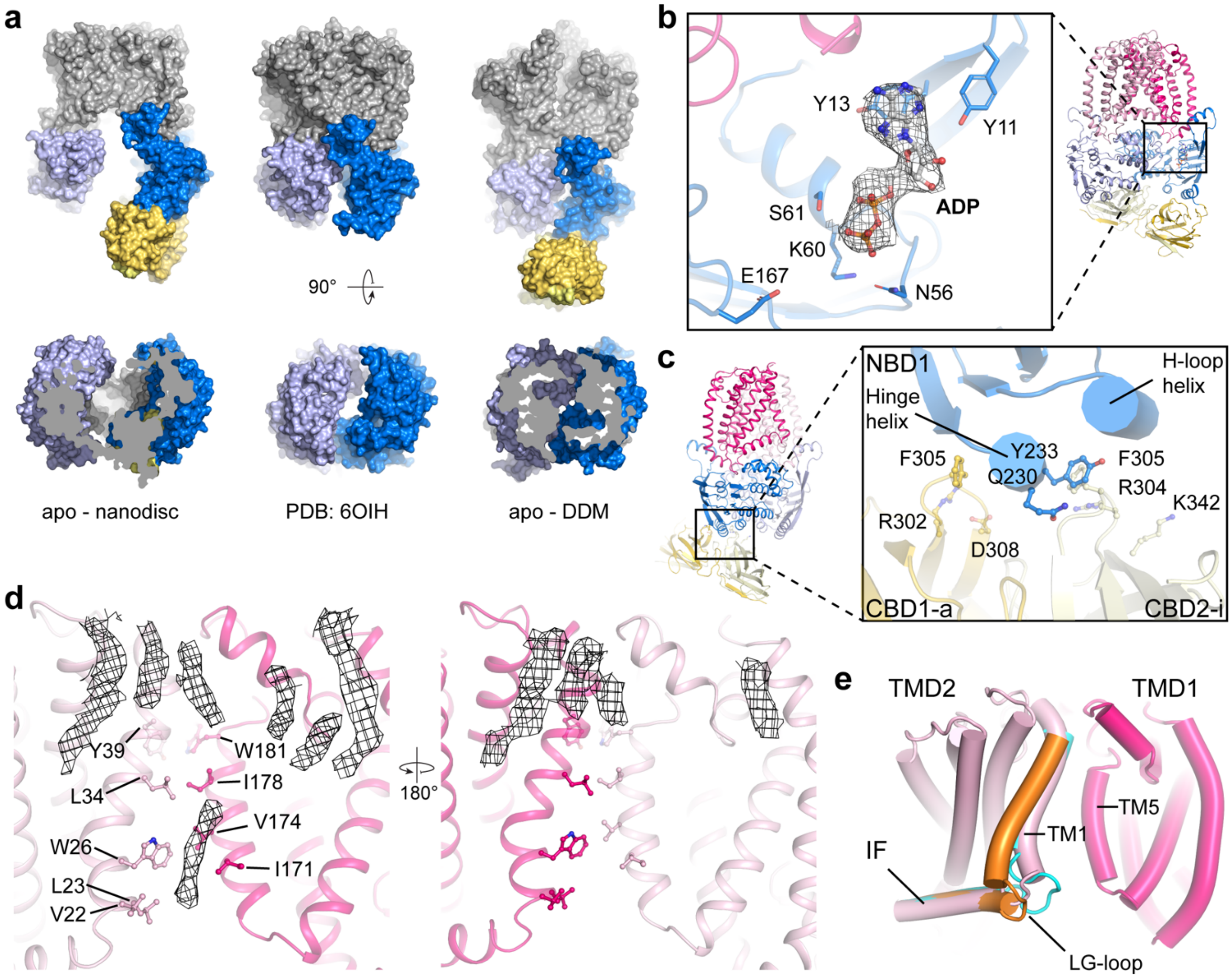

Supplementary Fig. 4| Map quality and structural contrasts between apo WzmWzt models. a, Comparison of the apo conformations of the transporter in nanodisc. **b**, ADP map from the ADP-bound WzmWzt transporter. **c**, Interactions between NBD1 and the CBD dimer in the ATP-bound WzmWzt (PDB ID: 7K2T). **d**, Lipid densities found in the nucleotide-free WzmWzt model (contoured at 5σ and shown as a black mesh). **e**, Conformation of the LG loop between the EM WzmWzt and X-ray WzmWztN structures**.**

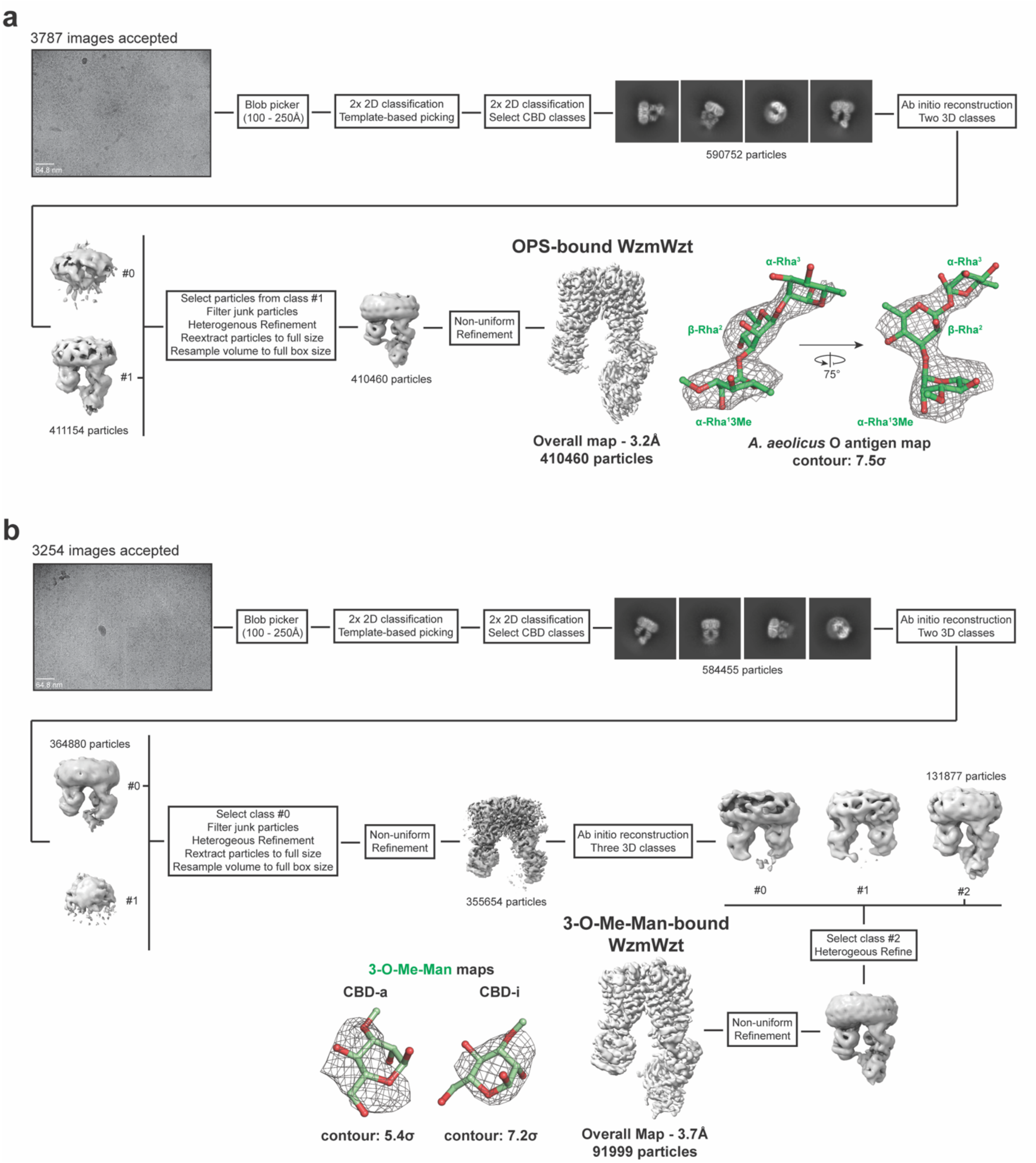

Supplementary Fig. 5| Cryo-EM data processing of (a) OPS and (b) 3-O-methyl-D-mannose bound WzmWzt.

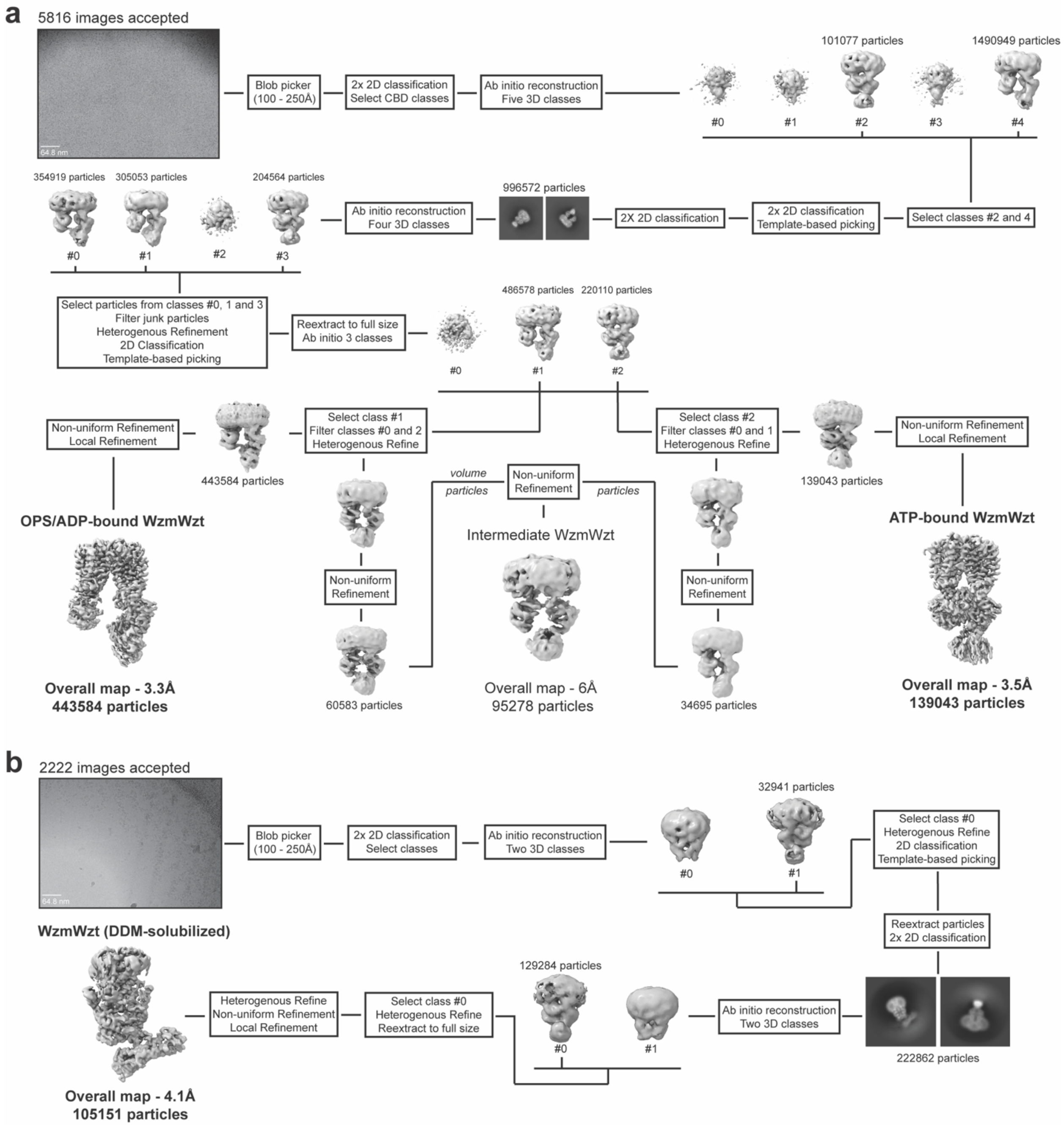

Supplementary Fig. 6| Cryo-EM data processing of WzmWzt under ATP hydrolysis conditions in the presence of O antigen (a) and nucleotide and substrate-free conditions *in surfo* (b).

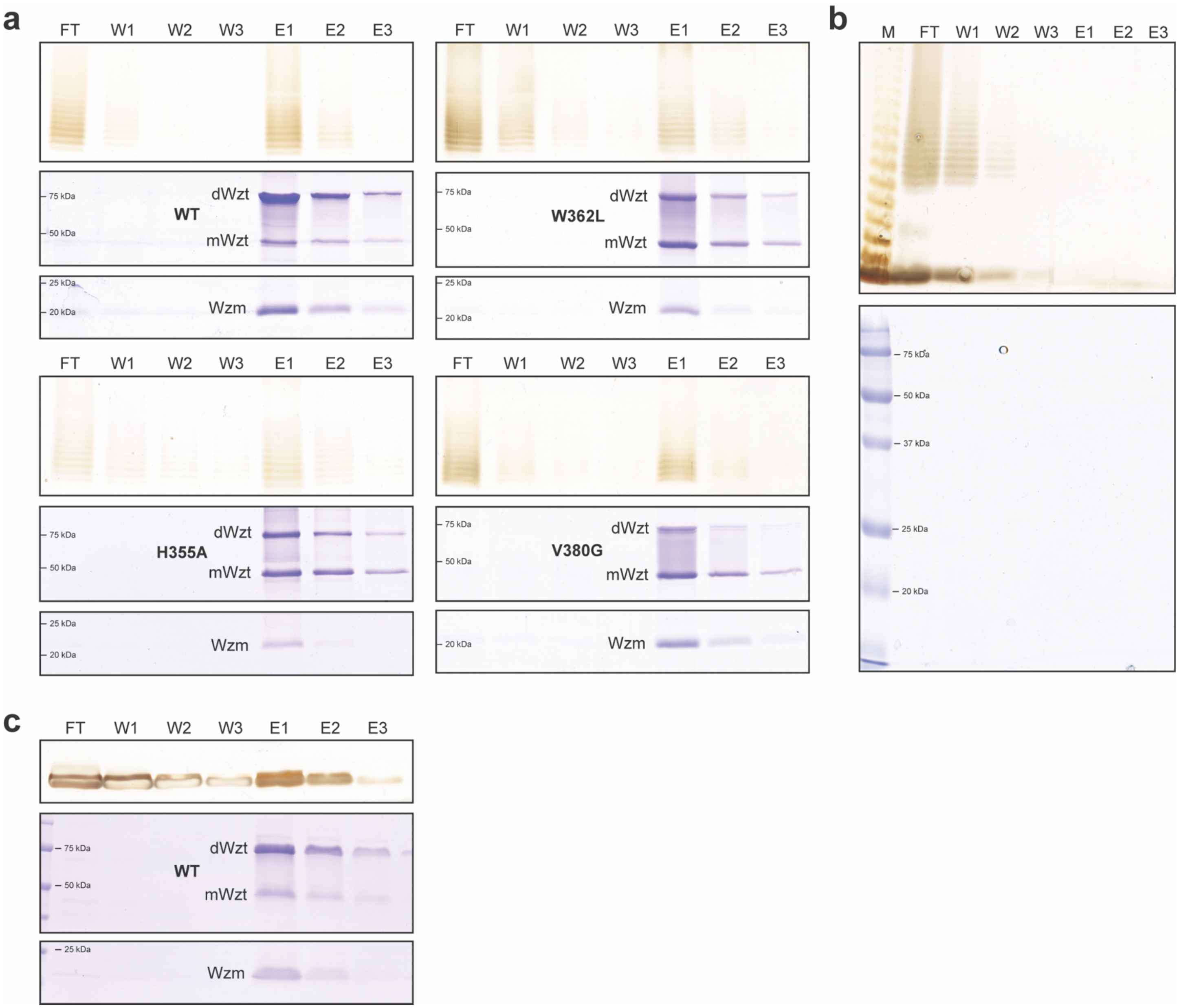

Supplementary Fig. 7| LPS pulldown using DDM-solubilized WzmWzt. a, *A. aeolicus* LPS pulldown examining binding against different DDM-solubilized WzmWzt mutant constructs. Top panel is silver stained LPS. Middle and bottom panels are Coomassie stained Wzt and Wzm, respectively. **b**, LPS pulldown control demonstrating *A. aeolicus* LPS does not bind to Ni-NTA resin. **c**, Kdo_2-lipid A pulldown examining binding against the wild type DDM-solubilized WzmWzt transporter.

SUPPLEMENTARY FIGURES UNCROPPED IMAGES

Supplementary Figure 7a

WzmWzt WT and AaLPS pulldown

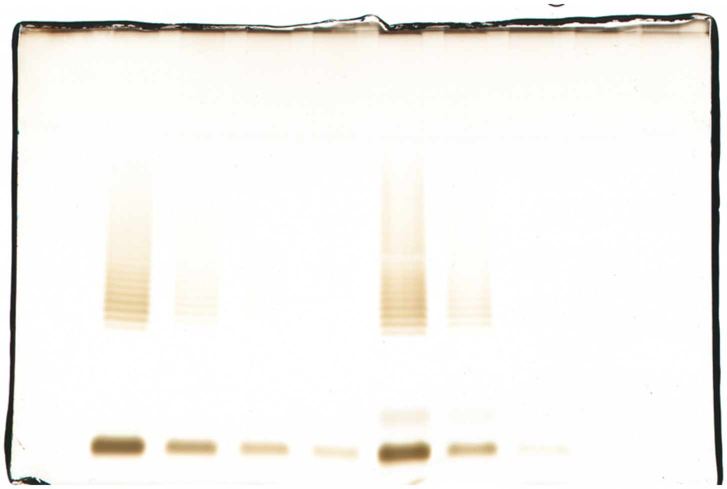

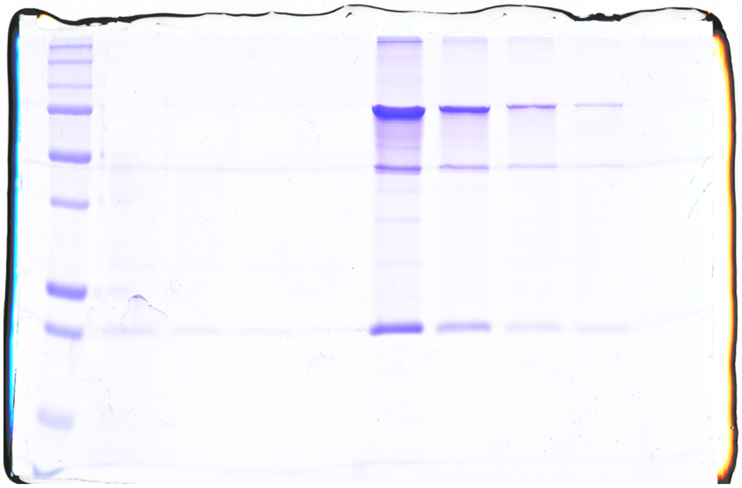

SUPPLEMENTARY FIGURES UNCROPPED IMAGES

Supplementary Figure 7a

WzmWzt H355A and AaLPS pulldown

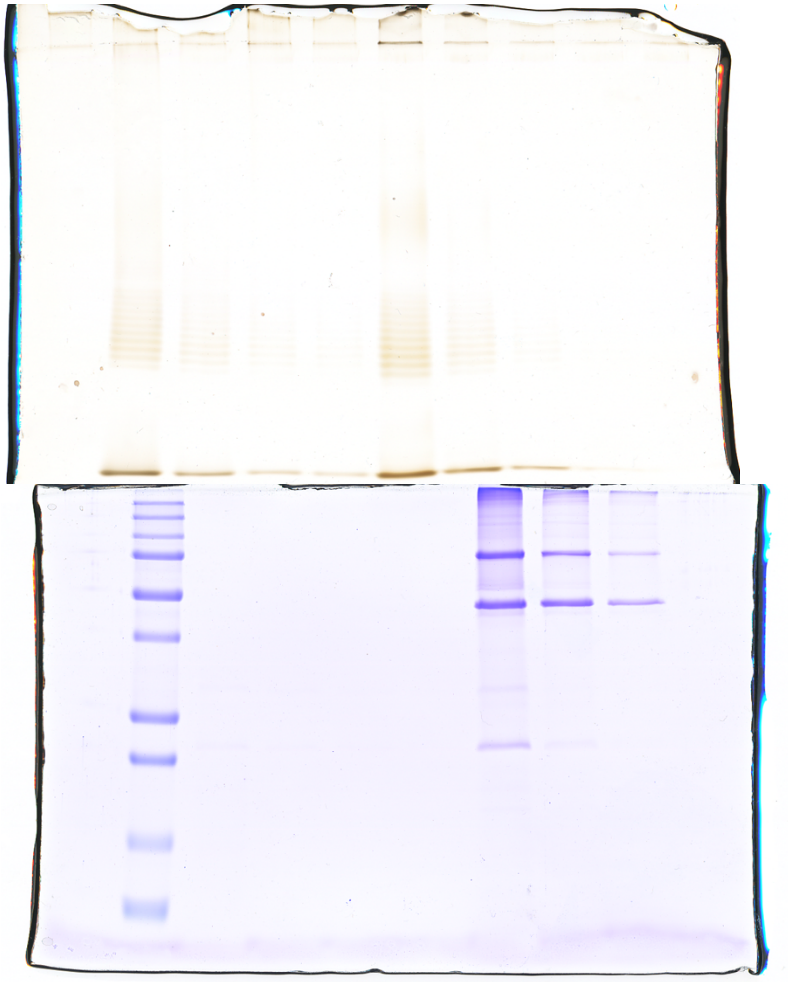

SUPPLEMENTARY FIGURES UNCROPPED IMAGES

Supplementary Figure 7a

WzmWzt W362L and AaLPS pulldown

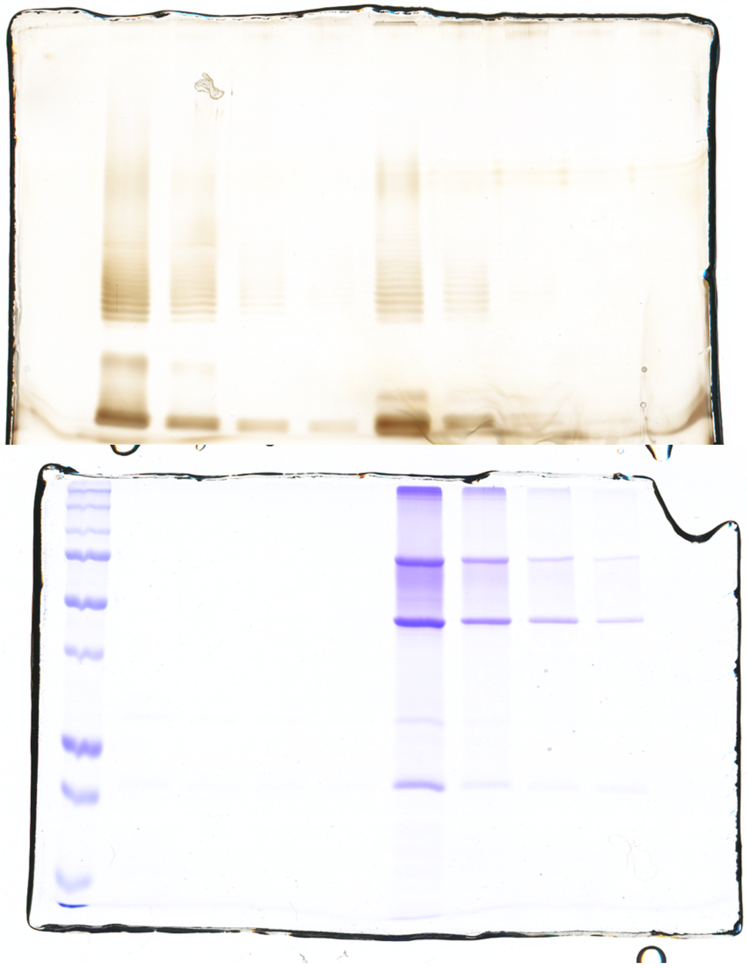

SUPPLEMENTARY FIGURES UNCROPPED IMAGES

Supplementary Figure 7a

WzmWzt V380G and AaLPS pulldown

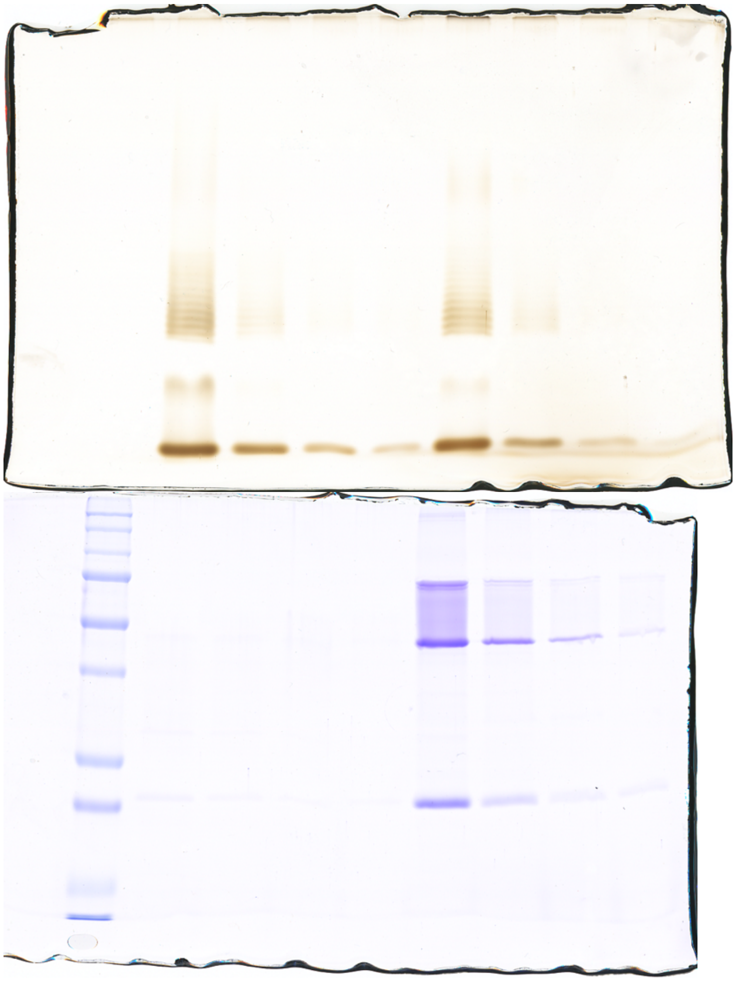

SUPPLEMENTARY FIGURES UNCROPPED IMAGES

Supplementary Figure 7b

AaLPS only, no WzmWzt

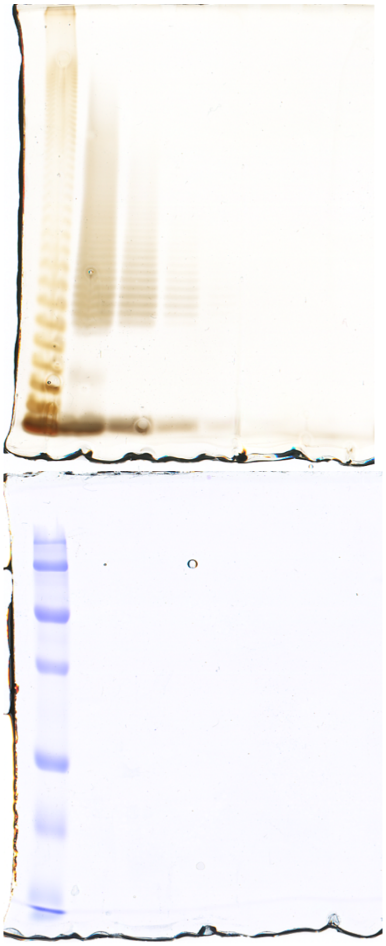

SUPPLEMENTARY FIGURES UNCROPPED IMAGES

Supplementary Figure 7c

WzmWzt and Kdo2-lipid A pulldown

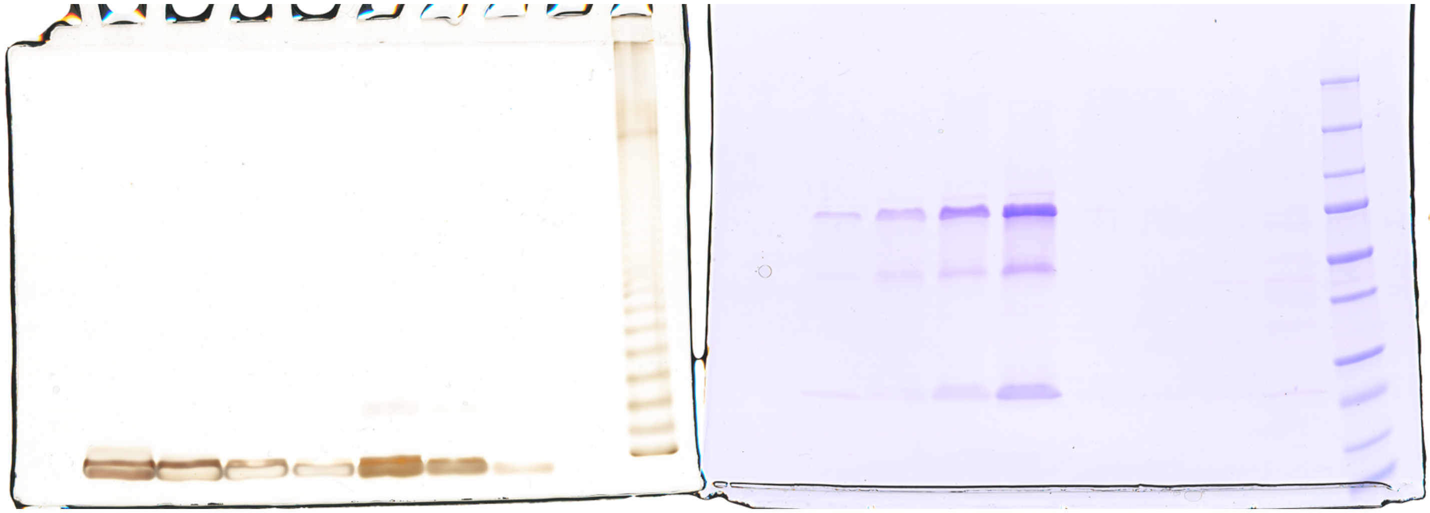

Note: Coomassie stained gel requires vertical flip
