# Supplementary material for: Molecular basis for polysaccharide recognition and modulated ATP hydrolysis by the O antigen ABC transporter
Source: Nat Commun. 2022 Sep 5;13:5226. doi: 10.1038/s41467-022-32597-2 (PMC9445017; doi:10.1038/s41467-022-32597-2)
Supplement: Supplementary file 3 — Description of Additional Supplementary Files [file 41467_2022_32597_MOESM3_ESM.pdf]

File name: Supplementary Movie 1

Description: Conformational changes of WzmWzt upon ATP binding.
