# Supplementary material for: Molecular basis for polysaccharide recognition and modulated ATP hydrolysis by the O antigen ABC transporter
Source: Nat Commun. 2022 Sep 5;13:5226. doi: 10.1038/s41467-022-32597-2 (PMC9445017; doi:10.1038/s41467-022-32597-2)

**MAIN TEXT UNCROPPED IMAGES**

Figure 1a

*Salmonella enterica* and *Aquifex aeolicus* VF5 LPS

**
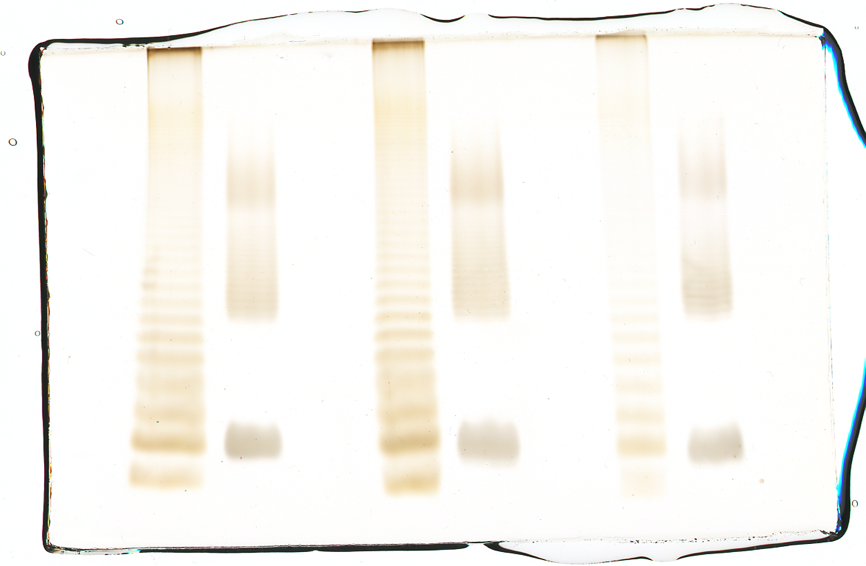
**

**MAIN TEXT UNCROPPED IMAGES**

Figure 6a

Wzt-CBD WT and AaLPS pulldown


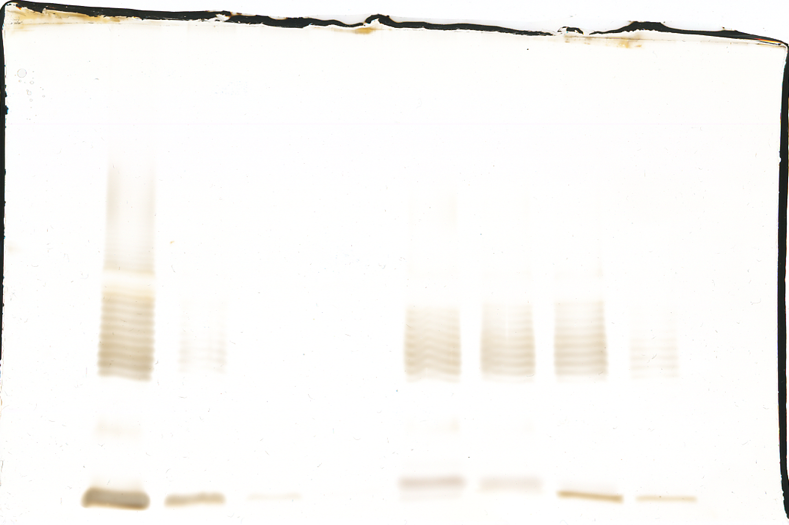


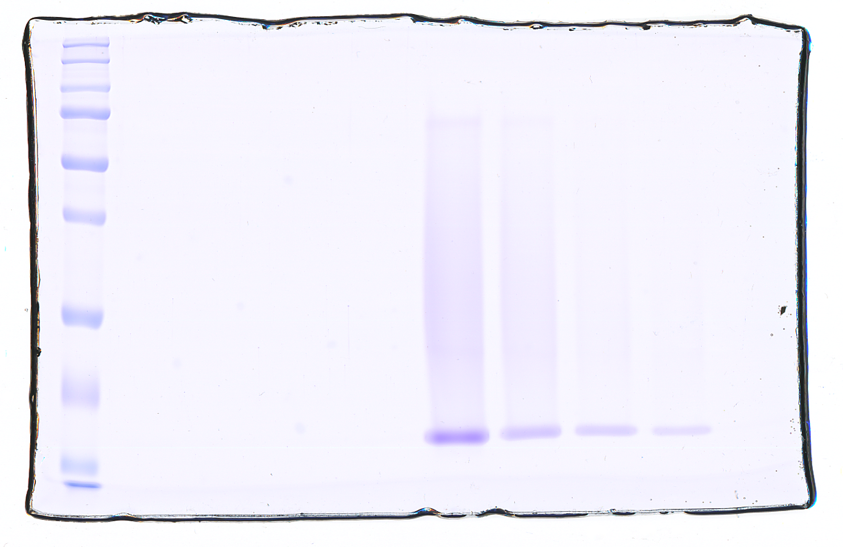


**MAIN TEXT UNCROPPED IMAGES**

Figure 6a

Wzt-CBD H355A and AaLPS pulldown


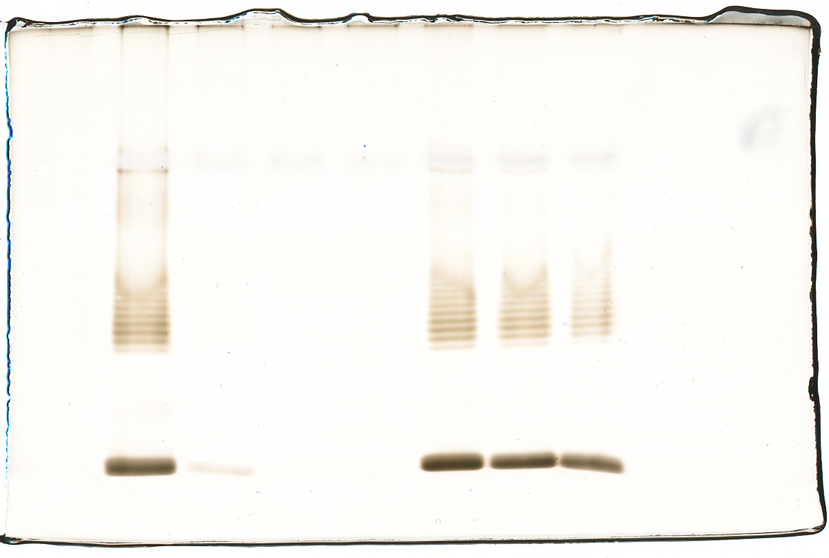


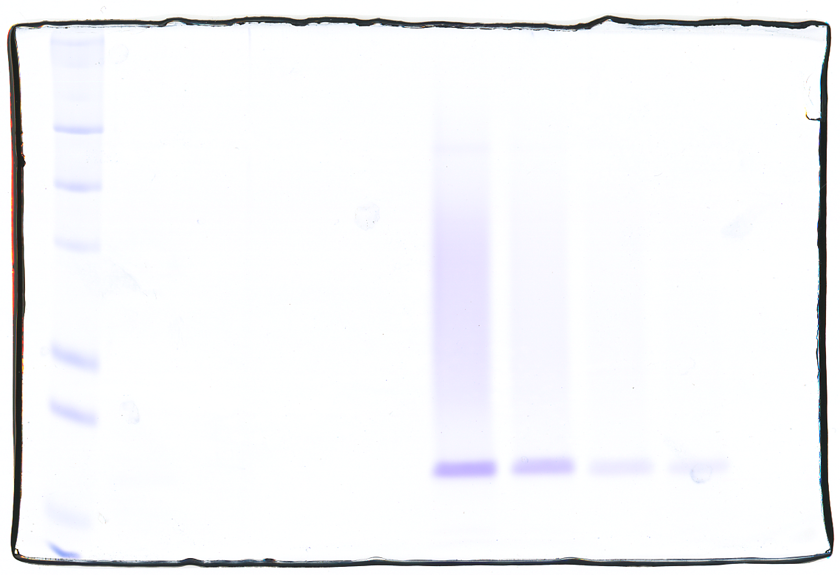


**MAIN TEXT UNCROPPED IMAGES**

Figure 6a

Wzt-CBD W362L and AaLPS pulldown


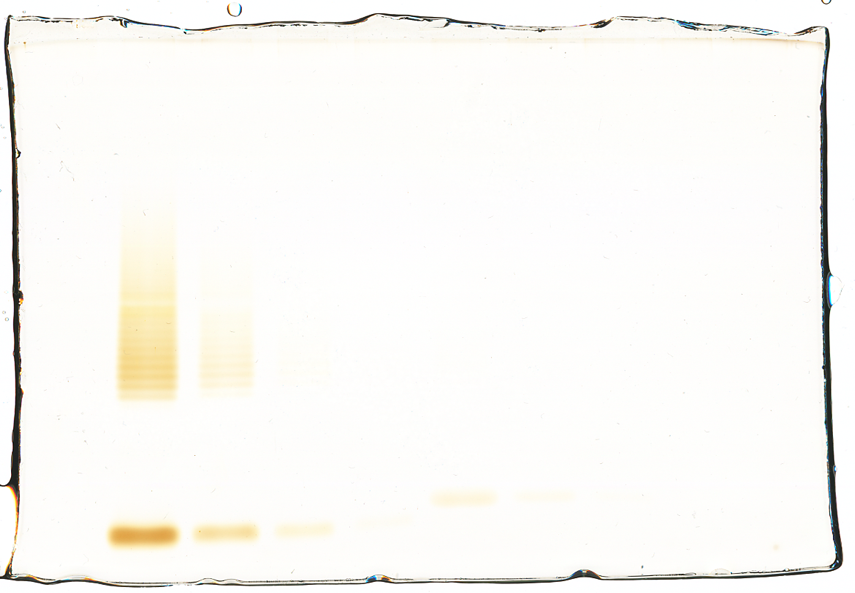


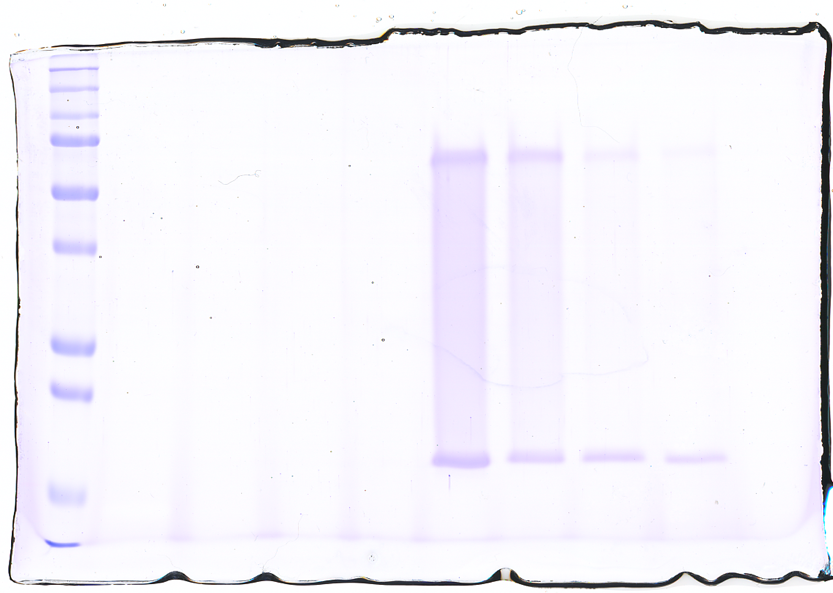


**MAIN TEXT UNCROPPED IMAGES**

Figure 6a

Wzt-CBD V380G and AaLPS pulldown


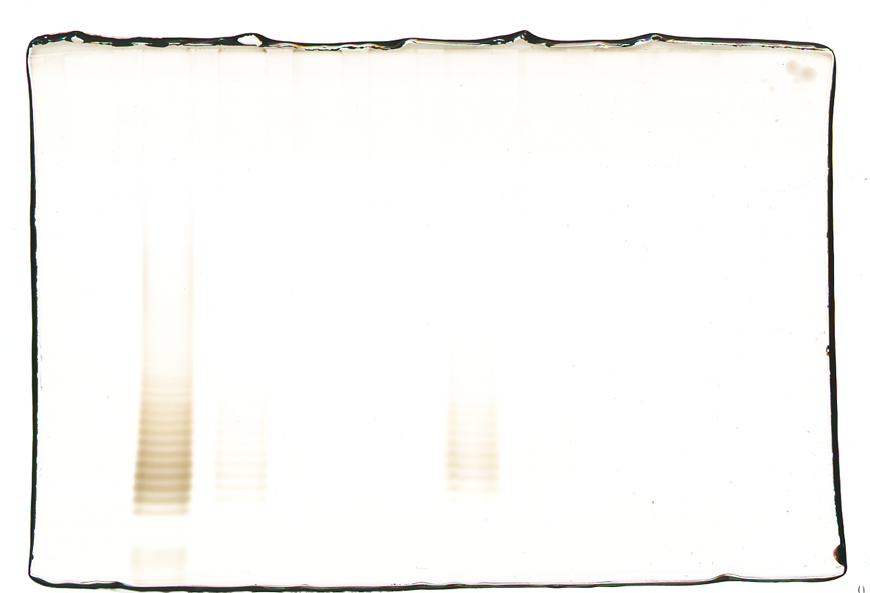


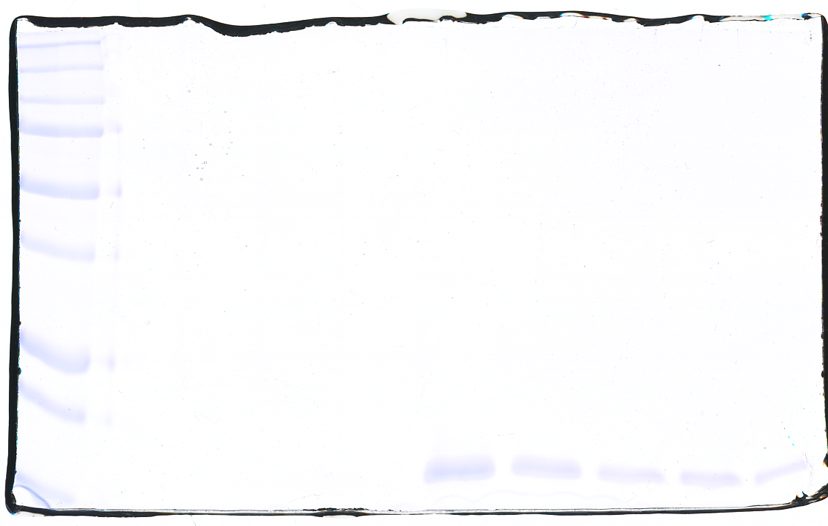

Supplement: Supplementary file 6 — Source Data [file 41467_2022_32597_MOESM6_ESM.zip › Uncropped images of gels.docx]
